# Teratosphaeria stem canker of *Eucalyptus*: two pathogens, one devastating disease

**DOI:** 10.1111/mpp.12758

**Published:** 2018-11-03

**Authors:** Janneke Aylward, Francois Roets, Leánne L. Dreyer, Michael J. Wingfield

**Affiliations:** ^1^ Department of Biochemistry, Genetics and Microbiology, Forestry and Agricultural Biotechnology Institute (FABI) University of Pretoria Pretoria 0002 South Africa; ^2^ Department of Conservation Ecology and Entomology Stellenbosch University, Private Bag X1 Matieland 7602 South Africa; ^3^ Department of Botany and Zoology Stellenbosch University, Private Bag X1 Matieland 7602 South Africa

**Keywords:** *Coniothyrium*, *Eucalyptus*, forestry, plantations, stem canker, *Teratosphaeria*

## Abstract

****Background**:**

*Teratosphaeria gauchensis *and *T. zuluensis* are closely related fungi that cause Teratosphaeria (previously Coniothyrium) stem canker disease on *Eucalyptus* species propagated in plantations for commercial purposes. This disease is present in many countries in which *Eucalyptus *trees are planted, and continues to spread with the international trade of infected plant germplasm.

**Taxonomy:**

Fungi, Ascomycota, Pezizomycotina, Dothideomycetes, Dothideomycetidae, Capnodiales, Teratosphaeriaceae, *Teratosphaeria.*

**Identification:**

The causal agents form dark masses of pycnidia that are visible on the surface of distinct stem cankers that typically form on young green stem tissues. Accurate diagnosis of the causal agents requires DNA sequence data.

**Host range:**

Nine species of *Eucalyptus *are known to be affected. Of these, *E. grandis *and its hybrids, which include some of the most important planting stock globally, appear to be particularly vulnerable.

**Disease symptoms:**

Small necrotic lesions develop on young green stem tissue. These lesions coalesce to form large cankers that exude gum. Epicormic shoots develop below the girdling canker and, in severe cases, trees die.

**Useful websites:**

Mycobank, https://www.mycobank.org; Publications of the Forestry and Agricultural Biotechnology Institute (FABI), https://www.fabinet.up.ac.za/index.php/journals.

## Introduction

Teratosphaeria stem canker is an important disease of *Eucalyptus *species planted outside their native range for the production of wood and wood products in countries with subtropical and tropical climates (Gezahgne *et al*., 2005; Wingfield *et al*., 1996). The disease is caused by two closely related species of Dothideomycete fungi in the genus *Teratosphaeria* (Order: Capnodiales; Family: Teratosphaeriaceae) (Crous *et al*., 2009a). Intriguingly, *T. gauchensis *and *T. zuluensis *cause Teratosphaeria stem canker with identical symptoms, independently in different parts of the world (Cortinas *et al*., 2006c).

Only the asexual states of *T. gauchensis *and *T. zuluensis* are known. Consequently, few morphological characters of taxonomic relevance are available to discriminate between the pathogens. Accurate identification and disease diagnosis relies entirely on DNA sequence comparisons (Roux *et al*., 2002). Microsatellite genotyping has also strongly influenced our understanding of the global prevalence of these two species. This review seeks to illustrate that these molecular genetic techniques have been indispensable in the study of these fungi and, further, that genomic tools will define the future understanding of their biology and management.

This pathogen profile reviews the available knowledge regarding Teratosphaeria canker and its causal agents, gathered subsequent to the first description of this disease more than two decades ago (Wingfield *et al*., 1996). The apparent and worrying ease with which Teratosphaeria canker has spread to plantations across the globe is discussed, and the growing threat of this disease to plantation forestry is also considered.

## A Globally Important Host

*Eucalyptus *is a large genus (>740 species) in the Myrtaceae, a family comprised predominantly of flowering trees and some shrubs (Rejmánek and Richardson, 2011). The genus is native to Australia and some surrounding islands in the Pacific, but many of its members thrive outside of their natural environment as a result of rapid growth and drought tolerance. Consequently, *Eucalyptus *species have been transported across the globe for use as shade, ornamental trees and wind barriers (Santos, 1998). Together with *Pinus *and some other tree genera, *Eucalyptus *trees have also become one of the main components in both Northern and Southern Hemisphere forest plantation operations (Carle and Holmgren, 2008; Del Lungo *et al*., 2006).

*Eucalyptus *trees are valuable not only for their timber, but also for non‐timber commodities, such as essential oils and honey (Rejmánek and Richardson, 2011). Globally, *Eucalyptus *plantations established for the production of wood products have increased in magnitude exponentially over the past century, and comprised an estimated 20 million hectares in 2008 (Rejmánek and Richardson, 2011). This equates to almost one‐quarter of the 92 million hectares of *Eucalyptus *in natural Australian forests (Australia's State of the Forests Report, 2013). The incredible success with which these trees have been grown outside their native range has largely been attributed to an initial escape from natural enemies (Wingfield, 2003). This disease‐free ‘honeymoon’ period has, however, been relatively short‐lived, and disease and pest problems have become an important constraint to the sustainability of *Eucalyptus* plantations globally (Burgess and Wingfield, 2017).

## Disease Impact

An unknown stem canker of *Eucalyptus grandis *was first reported in 1988 in a plantation in the vicinity of KwaMbonambi in Kwa‐Zulu Natal, South Africa (Wingfield *et al*., 1996). The pathogen was described as *Coniothyrium zuluense *(now known as *Teratosphaeria zuluensis*), and the disease was referred to as Coniothyrium (now Teratosphaeria) canker (Wingfield *et al*., 1996). This stem canker disease damaged large areas of *Eucalyptus *in the subtropical forestry regions of South Africa, necessitating the removal of numerous *E. grandis *stands (Cortinas *et al*., 2010).

**Figure 1:**
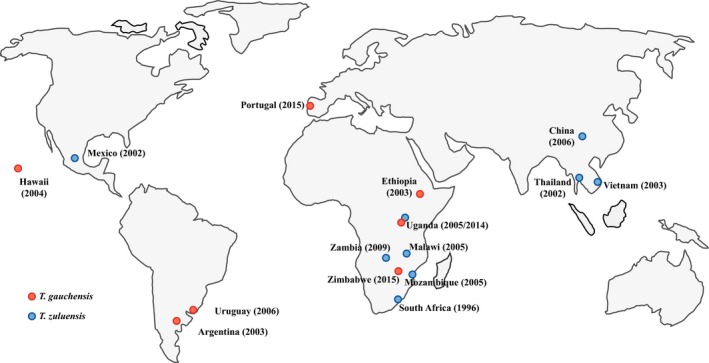
Global distribution of Teratosphaeria stem canker. For each country, the year of the first published disease report is shown in parentheses. Both *Teratosphaeria gauchensis *and *T. zuluensis *occur in Uganda. [Color figure can be viewed at wileyonlinelibrary.com]

Teratosphaeria canker was known only from South Africa until the early 2000s (Van Zyl *et al*., 2002b). Surveys of Australian plantations and natural forests have also failed to detect similar symptoms or pathogens, although closely related species causing leaf spots have been identified (Andjic *et al*., 2010; Crous *et al*., 2006). For this reason, it was initially hypothesized that the disease originated in South Africa, possibly as a result of a host shift (Slippers *et al*., 2005) from a closely related plant, probably one in the Myrtaceae (Wingfield *et al*., 1996). This would have been similar to the situation that has occurred with various other *Eucalyptus* canker pathogens in the Cryphonectriaceae (Nakabonge *et al*., 2006; Van der Merwe *et al*., 2010). However, Teratosphaeria canker has subsequently emerged in many other subtropical and tropical areas of the world in which *Eucalyptus *is planted for commercial forestry (Fig. 1). It now affects plantations in several Asian (Cortinas *et al*., 2006a; Gezahgne *et al*., 2003a; Van Zyl *et al*., 2002b), African (Gezahgne *et al*., 2005; Muimba‐Kankolongo *et al*., 2009; Roux *et al*., 2005) and South American (Cortinas *et al*., 2006c; Gezahgne *et al*., 2003a) countries, as well as in Mexico, Hawaii and Portugal (Cortinas *et al*., 2004; Roux *et al*., 2002; Silva *et al*., 2015).

Although trees die only in severe cases of the disease, the impact of Teratosphaeria canker on the forestry industry can be devastating. Stem cankers decrease the quality of timber to the extent that it cannot be used for construction (Gezahgne *et al*., 2003a). In addition, the wood is stained with red gum that exudes from the cankers (Gezahgne *et al*., 2003b; Old *et al*., 2003), decreasing the value of other timber products. The cost and quality of pulping are also negatively affected because the cankers hinder the de‐barking process.

## The Pathogen Emergence of a Cryptic Species

As the incidence of Teratosphaeria stem canker increased in plantations outside South Africa, a large collection of isolates became available to study the genetics of *T. zuluensis *(Cortinas *et al*., 2006b). Rather than enabling a population genetics study, genotypes of these isolates revealed two distinct taxa isolated from cankers on *Eucalyptus* that were indistinguishable based on symptoms (Cortinas *et al*., 2006c). The isolates accommodating individuals from southern Africa, Asia and Mexico represented *T. zuluensis*. In contrast, a second group emerged comprising isolates from South America, and these were provided with the name *T. gauchensis *(Fig. 1). Thus, two species, resulting in identical symptoms, occurred on the same hosts in different parts of the world.

The lack of clear morphological features for the Teratosphaeria canker pathogens has contributed to a series of changes in their taxonomy (Fig. S1, see Supporting Information). The few taxonomically useful morphological characteristics, such as conidial size and development, are inconspicuous and difficult to determine (Cortinas *et al*., 2006c; Crous *et al*., 2009a; Wingfield *et al*., 1996). The canker pathogen was identified as a Dothideomycete fungus and originally described in the genus *Coniothyrium *(Wingfield *et al*., 1996). DNA evidence later revealed a close relationship to *Mycosphaerella *in the order Capnodiales, causing it to be transferred sequentially to three traditionally asexual genera associated with the sexual genus *Mycosphaerella*, namely *Colletogleopsis*, *Kirramyces *and *Readeriella* (Andjic *et al*., 2007; Cortinas *et al*., 2006a; Crous *et al*., 2007). Finally, to escape the debates surrounding the appropriate asexual genus and in anticipation of the ‘one fungus one name’ principle (Hawksworth *et al*., 2011; Taylor, 2011), these *Eucalyptus*‐infecting fungi were transferred to the family Teratosphaeriaceae (Crous *et al*., 2007) and placed in the sexual genus *Teratosphaeria* (Crous *et al*., 2009a).

## Two Pathogens, One Disease

### Disease symptoms

**Figure 2:**
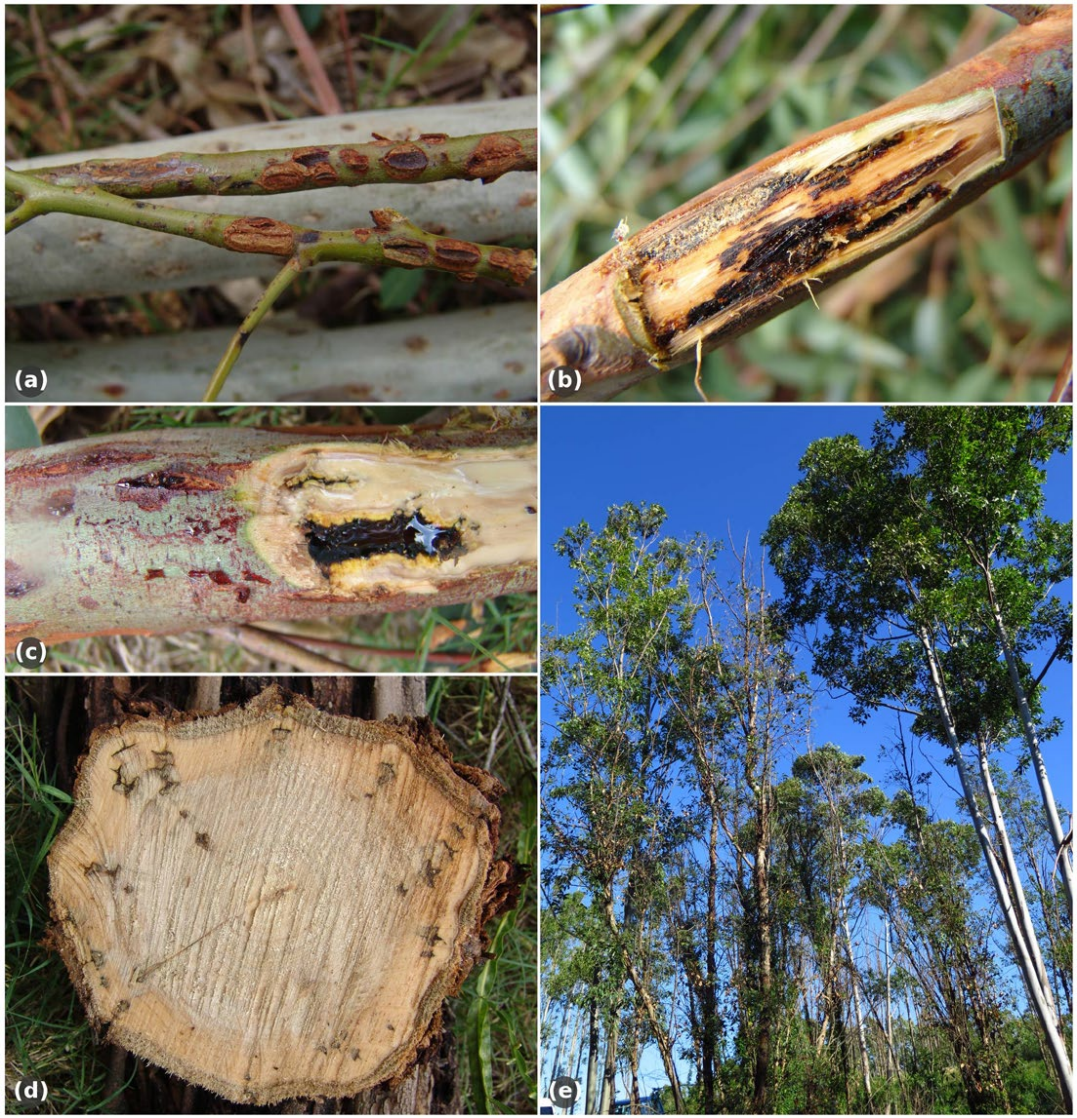
Symptoms of Teratosphaeria canker on *Eucalyptus*. (a, b) Necrotic lesions on young stems; (c) gum‐filled canker; (d) cross‐section of an infected stem; (e) resistant (right) and susceptible (left) *E. grandis *clones. Photographs: M. J. Wingfield. [Color figure can be viewed at wileyonlinelibrary.com]

*Teratosphaeria zuluensis *and *T. gauchensis* have very different geographical distributions (Fig. 1). It is intriguing that two allopatric pathogens cause indistinguishable disease symptoms independently. New infections typically occur during spring, after which Teratosphaeria canker is first visible as small (2–5 mm) necrotic lesions on *Eucalyptus* stems (Wingfield *et al*., 1996) (Fig. 2), with young stem tissues particularly vulnerable (Old *et al*., 2003). These lesions become elliptical as they grow in size (Cortinas *et al*., 2006c), penetrate the vascular cambium (Old *et al*., 2003) and eventually merge with neighbouring lesions to form cankers filled with gum, also known as kino pockets (Wingfield *et al*., 1996; Fig. 2). Stem malformation typically ensues and the bark covering these cankers often cracks vertically, creating a ‘cat‐eye’ appearance and causing the gum to exude (Cortinas *et al*., 2006c). In the case of severe infections on susceptible clones, cankers girdle the stems, epicormic shoots develop and the tops of the trees die (Old *et al*., 2003; Wingfield *et al*., 1996).

A study by Van Zyl *et al*. (2002a) showed that the pathogenicity of different isolates of *T. zuluensis *collected from the same area of disease occurrence varied considerably. The majority of isolates (>70%) did not give rise to noticeable lesions on 6‐month‐old *E. grandis *seedlings and appeared to be non‐pathogenic. The remainder of the isolates produced lesions ranging between 15 and 61 mm, and the most virulent isolates (causing the longest lesions) also induced swelling and necrosis of stem tissue around the inoculation site.

### Pathogen identification

*Teratosphaeria zuluensis *and *T. gauchensis *grow within host tissue, but are visible on the surface of cankers as dark pycnidia, producing large numbers of dark, single‐celled conidia (Wingfield *et al*., 1996). During moist conditions, black conidial tendrils extend from the canker surfaces (Cortinas *et al*., 2006c), but sexual structures of *T. zuluensis *and *T. gauchensis* have never been observed in the field or in culture (Wingfield *et al*., 1996).

Morphological distinction between *T. zuluensis *and *T. gauchensis *is not possible with any reasonable level of confidence. The two species have overlapping conidial lengths, although *T. gauchensis *conidia tend to be slightly longer and narrower (Silva *et al*., 2015). The conidia of *T. gauchensis *develop from polyphialidic conidiogenous cells, whereas *T. zuluensis* has percurrently proliferating monophialidic conidiogenous cells and the culture morphology is similarly variable for both species (Cortinas *et al*., 2006a, 2006c ; Van Zyl *et al*., 2002b) (Fig. S2, see Supporting Information). Culture margins are grey or white and may be smooth or irregular. The centres are typically varying shades of olive or grey that darken with age. From below, cultures range from dark green to rust/brown and may have lighter white or olivaceous bands at the margins.

Molecular data provide the only reliable means to identify the species of *Teratosphaeria* causing *Eucalyptus* cankers. Phylogenetic studies group the two species into closely related clades with 26 fixed polymorphisms between them in the internal transcribed spacer (ITS), β‐tubulin, elongation factor 1‐α (EF1‐α) and ATP6 genes (Cortinas *et al*., 2004; Gezahgne *et al*., 2005; Van Zyl *et al*., 2002b). A 20‐base‐pair fragment in the EF1‐α intron is absent in *T. gauchensis*, but present in *T. zuluensis*, providing a useful marker for distinction between the species.

Intraspecific phylogenetic structure exists in both pathogens, but is especially common in *T. zuluensis *(Cortinas *et al*., 2006c; Jimu *et al*., 2014; Silva *et al*., 2015). *Teratosphaeria zuluensis* isolates from some localities form well‐supported subclades, but isolates from localities with moderate to high genetic diversities, such as South Africa and China, intersperse with those from other locations (Chen *et al*., 2011; Cortinas *et al*., 2010). These subclades are not substantiated by biological information and only one study has reported a difference in the temperature range and pathogenicity of a well‐supported group of *T. zuluensis *isolates from Thailand (Van Zyl *et al*., 2002b).

The lack of clear geographical boundaries and biological information between intraspecific clades in *T. zuluensis *and *T. gauchensis* precludes the description of additional species. However, considering the intraspecific variability, it may be relevant to refer to these species as species complexes: a group of monophyletic isolates with significant genetic differences that are not detected by standard barcoding or diagnostic procedures (Chen *et al*., 2016). Further distinction only becomes necessary if clinical, diagnostic or ecological relevance can be attached to different isolates in the complex (Chen *et al*., 2016), which is presently not the case for *T. zuluensis *or *T. gauchensis*.

### Ecological factors

The *Teratosphaeria *stem canker pathogens grow very slowly in culture, taking 6 weeks at 25 °C to reach a diameter of 40–50 mm (Cortinas *et al*., 2006c). This poor growth probably indicates a reliance on living host tissue, and therefore a biotrophic lifestyle (Wingfield *et al*., 1996). The two species differ in the extremes of their temperature range: *T. gauchensis *appears to tolerate lower temperatures (*c*. 10 °C) better than *T. zuluensis*, which, in turn, grows at 35 °C where *T. gauchensis *growth shows a marked decrease (Silva *et al*., 2015). At their optimum range of 20–25 °C, *T. zuluensis *achieves a larger maximum diameter than *T. gauchensis* (Cortinas *et al*., 2006c).

The optimum temperature for growth of *T. zuluensis* and *T. gauchensis* might explain why canker outbreaks occur in subtropical and tropical plantations. Countries such as South Africa and Ethiopia have *Eucalyptus *plantations in both subtropical and temperate regions, yet Teratosphaeria canker is damaging only in subtropical areas (Gezahgne *et al*., 2005; Wingfield *et al*., 1996). Recently, however, *T. gauchensis *has been identified from Teratosphaeria canker symptoms on *E. globulus* in Portugal, a country that has a moderate Mediterranean climate (Silva *et al*., 2015). These authors also noted that *T. gauchensis *isolates from the cooler northern part of the country were less tolerant to high temperatures (35 °C), implying that the pathogen may be adapting to the cooler environment.

*Teratosphaeria gauchensis *has been isolated from seven different *Eucalyptus *plantation species (excluding hybrids) and *T. zuluensis *from four (Table 1). Currently, their only shared hosts are *E. camaldulensis *and *E. grandis *(Jimu *et al*., 2015; Van Zyl *et al*., 2002b), two of the most widely planted *Eucalyptus* species in tropical and subtropical regions. Ironically, *E. grandis *and its hybrids appear to be highly susceptible to Teratosphaeria canker. The remainder of the known host species, especially *E. cloeziana*, *E. paniculata* and *E. propinqua*, are less common (FAO, 1981). Similarly, although *E. globulus *is widely planted, it is typically planted in temperate climates in which the stem canker pathogens do not flourish (FAO, 1981). Therefore, it is likely that the different hosts of *T. gauchensis *and *T. zuluensis *reflect the distribution of *Eucalyptus *species in global plantations rather than true host preferences of these fungi.

**Table 1:** *Eucalyptus *species known to host the stem canker pathogens *Teratosphaeria gauchensis *and *T. zuluensis*.

*Teratosphaeria gauchensis*				*Teratosphaeria zuluensis*		
*Eucalyptus* host	Country	References		*Eucalyptus* host	Country	References
*E. camaldulensis* *	Ethiopia, Zimbabwe	Gezahgne *et al*. (2003b), Jimu *et al*. (2015)		*E. camaldulensis* *	Thailand	Van Zyl *et al*. (2002b)
*E. grandis* *	Argentina, Hawaii, Uganda, Uruguay, Zimbabwe	Cortinas *et al*. (2004, 2006b), Gezahgne *et al*. (2003a, 2005), Jimu *et al*. (2015)		*E. grandis* *	Malawi, Mozambique, Mexico, South Africa, Uganda, Zambia	Jimu *et al*. (2014), Muimba‐Kankolongo *et al*. (2009), Roux *et al*. (2002), 2005), Wingfield *et al*. (1996)
*E. globulus*	Portugal, Uruguay	Pérez *et al*. (2009), Silva *et al*. (2015)		*E. cloeziana*	Zambia	Chungu *et al*. (2010), Muimba‐Kankolongo *et al*. (2009)
*E. maidenii*	Uruguay	Pérez *et al*. (2009)		*E. urophylla*	Vietnam, China	Cortinas *et al*. (2006a), Gezahgne *et al*. (2003a)
*E. paniculata*	Zimbabwe	Jimu *et al*. (2015)				
*E. propinqua*	Zimbabwe	Jimu *et al*. (2015)				
*E. tereticornis*	Uruguay	Pérez *et al*. (2009)				

* Hosts infected by both *T. gauchensis *and *T. zuluensis*.

## Relationship to *Eucalyptus *Leaf Pathogens

**Figure 3:**
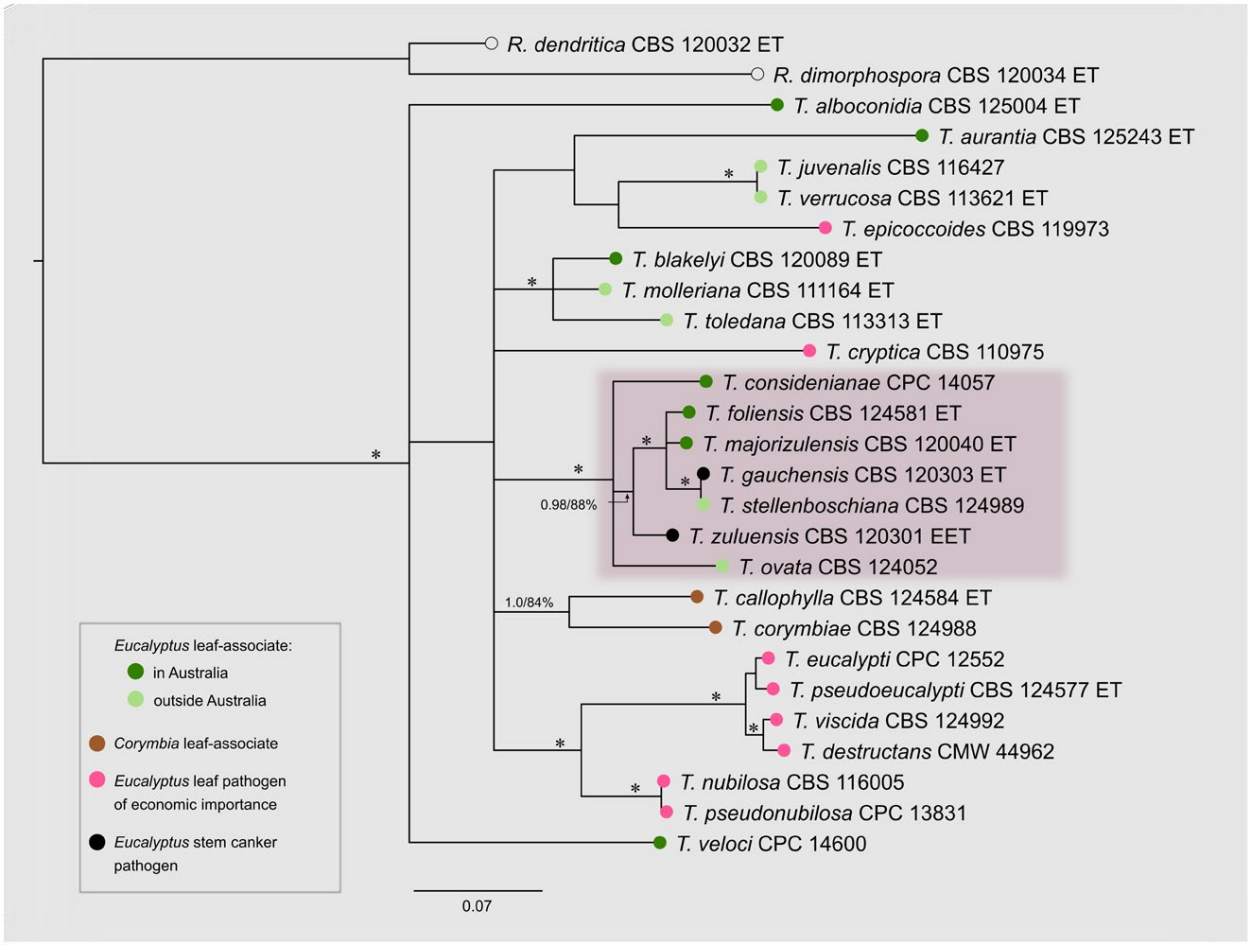
Bayesian 50% majority‐rule consensus tree of the concatenated β‐tubulin and elongation factor 1‐α (EF‐1α) genes of 25 *Teratosphaeria *species and two *Readerialla *outgroups. Asterisks (*) indicate branches with a Bayesian posterior probability of 1.00 and >95% maximum likelihood bootstrap; ET, ex‐type; EET, ex‐epitype. The clade containing the stem canker pathogens is highlighted. [See Text S1 (Supporting Information) for detailed methods and GenBank^®^ accession numbers.] [Color figure can be viewed at wileyonlinelibrary.com]

*Teratosphaeria zuluensis *and *T. gauchensis* are the only known stem canker pathogens in a clade of species predominantly associated with *Eucalyptus *leaves (Crous *et al*., 2009a; Quaedvlieg *et al*., 2014) (Fig. 3). The majority of these leaf associates cause leaf spots of minor importance. However, some, such as *T. destructans*, *T. cryptica*, *T. nubilosa *and *T. pseudoeucalypti*, are responsible for major economic losses to the forestry industry (Burgess and Wingfield, 2017; Cândido *et al*., 2014; Hunter *et al*., 2011). Surprisingly, DNA evidence has shown that the two stem canker pathogens are each more closely related to *Eucalyptus *leaf‐associated species than they are to each other (Quaedvlieg *et al*., 2014). Based on the ITS and EF‐1α genes, *T. gauchensis *groups closest to *T. majorizuluensis*, *T. foliensis *and *T. stellenboschiana* (Quaedvlieg *et al*., 2014; Silva *et al*., 2015) (Fig. 3). With the exception of *T. stellenboschiana*, discovered in Stellenbosch, South Africa, these leaf pathogens were all described from eastern Australia (Andjic *et al*., 2010; Crous *et al*., 2009b, 2006; Summerell *et al*., 2006).

It seems unlikely that two species causing the same disease symptoms are not each other’s closest relatives (Gezahgne *et al*., 2005; Old *et al*., 2003). An association with *Eucalyptus *leaves also appears to be ancestral, as other species in the phylogenetic clade are all leaf associates (Crous *et al*., 2009a) (Fig. 3). Pathogenicity to *Eucalyptus* stems would, consequently, have had to evolve separately in *T. zuluensis *and *T. gauchensis*. In fact, *T. gauchensis* has been isolated from leaf specks on *E. maidenii *and *E. tereticornis *(Pérez *et al*., 2009), and *T. zuluensis *has been isolated as an *E. grandis *leaf endophyte (Marsberg *et al*., 2014). These results suggest that they retain some affinity for leaves and support the notion that they could have emerged from leaf‐associated ancestors.

## Possible Origins

Some diseases of *Eucalyptus*, such as Cryphonectria canker and Myrtle rust, have emerged from pathogens moving to exotic *Eucalyptus *plantations from native plant hosts (Glen *et al*., 2007; Nakabonge *et al*., 2006). Others are caused by pathogens that are known to have been introduced from the native range of *Eucalyptus* (Wingfield, 2003; Wingfield *et al*., 2008). The latter pathogens are typically of little consequence in natural *Eucalyptus *forests, but can become critically important in commercial monoculture stands (Burgess and Wingfield, 2002; Park *et al*., 2000).

### Clues from population genetics studies

Population genetics studies do not support a South African origin for *T. zuluensis*, as originally hypothesized by Wingfield *et al*. (1996). Isolates from China and Malawi have greater gene and genotypic diversities than South African *T. zuluensis *populations (Chen *et al*., 2011; Cortinas *et al*., 2010), incongruent with the higher genetic diversity expected in the native range of a species (Hunter *et al*., 2008; McDonald, 1997). *Teratosphaeria zuluensis* populations elsewhere in Africa have low genetic diversity, consistent with recent introductions (Dlugosch and Parker, 2008; Jimu *et al*., 2016a). All *T. zuluensis* populations analysed thus far have been predominantly clonal with strong population differentiation.

The population genetic structure of *T. gauchensis *in South America does not necessarily rule out a host jump from a native plant. South American populations of *T. gauchensis*, distributed between Argentina and Uruguay are surprisingly cohesive and diverse (Cortinas *et al*., 2011). Surveys of native Myrtaceae in Uruguay, however, identified only one species of *Teratosphaeria *that also occurs on *Eucalyptus*, and concluded that this species jumped from *Eucalyptus *onto the native plant and not the other way around (Pérez *et al*., 2013). Similarly, the five other members of the Mycosphaerellaceae and Teratosphaeriaceae that were identified on native Myrtaceae in the study of Pérez *et al*. (2013) are known *Eucalyptus *associates and do not represent a shift from native plants to *Eucalyptus*.

In Africa, *T. gauchensis *populations in different countries are not structured and share genotypes, but genetic diversity is much lower than in South America (Jimu *et al*., 2016b). The genetic diversity and genotype identities in Zimbabwe implicate this country as the source of *T. gauchensis* introductions into Uganda and Ethiopia (Jimu *et al*., 2016b). The African condition reflects the anthropogenic movement of *T. gauchensis *between forestry plantations. Therefore, it is possible that the cohesive South American populations are a result of multiple introductions of the pathogen and high levels of trade in plant material between the different regions.

### Australia: the source?

The majority of *Eucalyptus *leaf diseases, caused by species of *Mycosphaerella *and *Teratosphaeria*, are believed to have originated in Australia. Population genetics studies have confirmed high genetic diversities in eastern Australia for *T. nubilosa *(Hunter *et al*., 2008; Pérez *et al*., 2012) and *T. epicoccoides *(Taole *et al*., 2015). *Teratosphaeria* *nubilosa *is devastating in plantations, both in Australia and globally (Hunter *et al*., 2009), and *T. epicoccoides *has been associated with severe outbreaks in an Australian plantation (Carnegie, 2007), but these species do not cause significant damage in natural forests, probably because of the long evolutionary association with their *Eucalyptus *hosts. The species richness of *Mycosphaerella *and *Teratosphaeria *species in natural *Eucalyptus *forests appears to be considerable, as surveys typically identify several new species (e.g. Andjic *et al*., 2010, Crous *et al*., 2009b, Summerell *et al*., 2006). Because of the numerous *Teratosphaeria* leaf pathogens known from Australia, the many that are probably still unknown and the minor symptoms that often occur on *Eucalyptus *trees in their natural environment, it is plausible that *T. zuluensis* and *T. gauchensis* also occur in these forests, but in the absence of disease symptoms.

**Figure 4:**
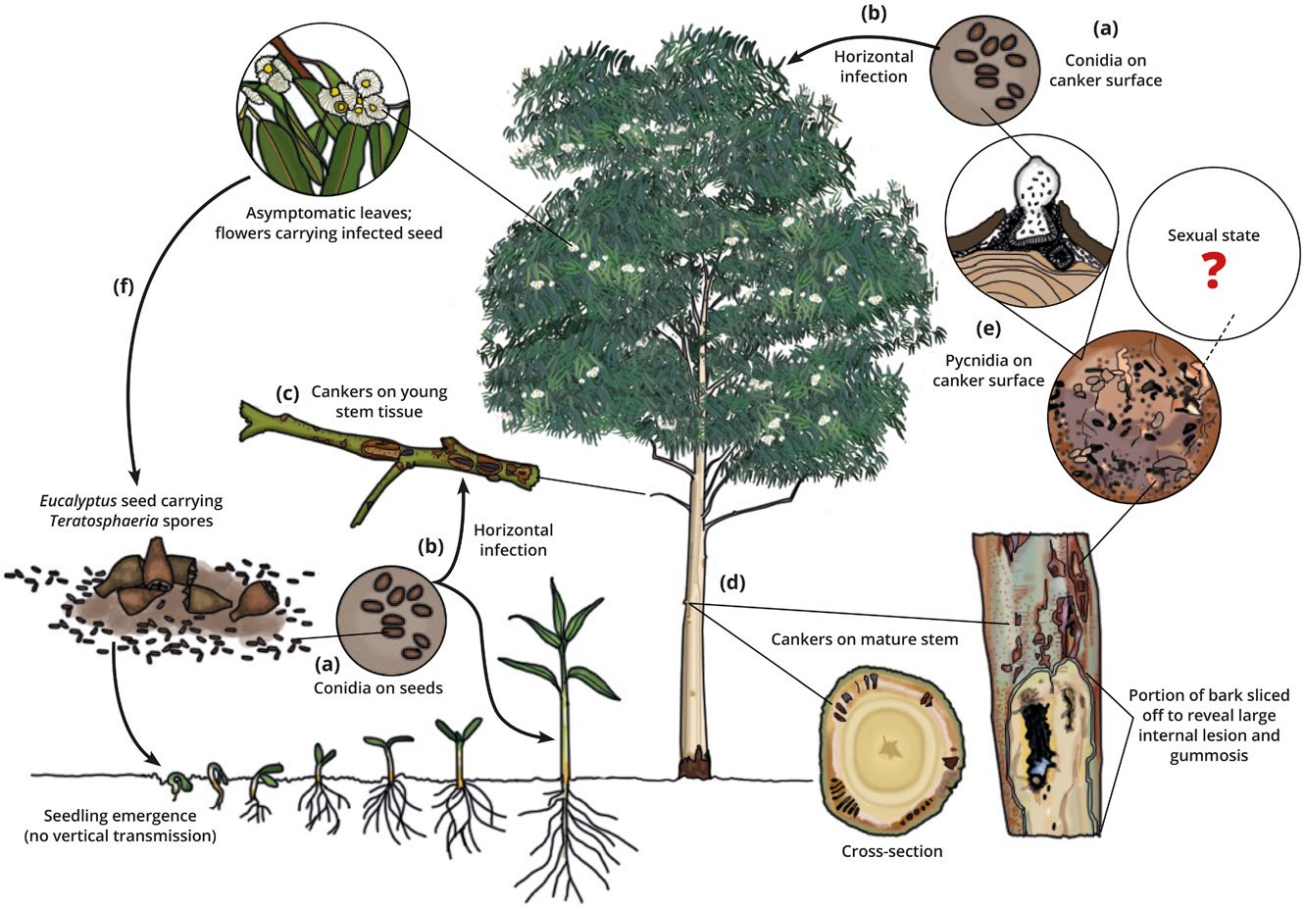
Putative infection cycle of *Teratosphaeria gauchensis *and *T. zuluensis *on a *Eucalyptus *host. (a) Asexual conidia are produced on the surface of cankers and moribund seed capsules, but do not enter plants vertically. (b) Conidia infect germinating seedlings and healthy plant tissues horizontally (Jimu *et al*., 2016c), and appear to remain dormant until susceptible trees reach a stage at which cankers are able to develop, typically at about 6 months of age. (c) Necrotic lesions appear and (d) eventually develop into gum‐filled cankers that extend into the vascular cambium. (e) Fungal pycnidia often break through the surface of the young stem or bark, but the sexual states of these fungi have not been identified. (f) These fungi have been identified in mature *Eucalyptus *leaves and the seed and seed capsules of infected trees. Seed dispersal also appears to be the means by which these fungi have moved globally. [Color figure can be viewed at wileyonlinelibrary.com]

High‐throughput sequencing (Kemler *et al*., 2013) and culture‐dependent studies (Marsberg *et al*., 2014) have confirmed the presence of *T. zuluensis *as an *E. grandis *endophyte. The *E. grandis *trees analysed were from a South African plantation affected by Teratosphaeria canker. Although they do not support an Australian origin, these studies indicate that *T. zuluensis *can exist as an endophyte (Fig. 4). Similarly, Jimu *et al*. (2016c) showed that *T. zuluensis* occurs on the seed and seed capsules of *E. grandis *trees displaying symptoms of Teratosphaeria canker. This is particularly significant, as the initial outbreak in South Africa was predominantly from seed‐derived *E. grandis *(Wingfield *et al*., 1996), and the study thus provided the first experimental evidence of *Teratosphaeria* species moving globally with seed consignments. Jimu *et al*. (2016c), however, also noted that infected seed does not yield infected seedlings, suggesting that horizontal rather than vertical pathogen transmission causes disease (Fig. 4).

The spread of *Eucalyptus *across plantations globally, and the diseases that tend to ensue, provides strong evidence that pests and pathogens are unwittingly moved around with *Eucalyptus *plant material (Jimu *et al*., 2016a, 2016c; Taole *et al*., 2015). *Teratosphaeria *species have no known association with animal vectors, and the population genetics of pathogenic *Teratosphaeria *species studied thus far implies limited dispersal and gene flow. The different geographical distributions of *T. zuluensis *and *T. gauchensis* complicate the hypothesis that these species are natural *Eucalyptus *associates, as a common endophytic origin should have resulted in concurrent introductions. However, the numerous fungi naturally associated with *Eucalyptus* and the intraspecific variability of *T. zuluensis *and *T. gauchensis* imply that a diverse suite of potential pathogens resides within natural *Eucalyptus *forests. Combined with the frequent movement of *Eucalyptus *germplasm from Australia to and between other countries, multiple introductions of *Eucalyptus *endophytes may have facilitated the emergence of different pathogens in the different environments. Current evidence therefore suggests that the Teratosphaeria canker pathogens occur naturally with *Eucalyptus* and have been co‐introduced into plantations with the germplasm (plants or seeds) of their hosts.

## Putative Infection Cycle

The life cycles of the stem pathogens *T. gauchensis *and *T. zuluensis *have never been elucidated. However, evidence gathered during the course of the past two decades provides a likely scenario that emerges in *Eucalyptus *plantations (Fig. 4). Although they are known only as pathogens of stem tissues, both *T. gauchensis *and *T. zuluensis* have been isolated from healthy *Eucalyptus *leaves (Marsberg *et al*., 2014; Pérez *et al*., 2009). In addition, a metagenomics study has revealed the presence of *T. zuluensis* in the seeds and seed capsules of trees suffering from Teratosphaeria canker (Jimu *et al*., 2016c). Seedlings grown from clean seed did not contain the pathogen, suggesting that it is not vertically transferred from seed to plant, but rather that seedlings are infected horizontally after they begin to grow. Infection of the green tissues of *Eucalyptus *trees most probably occurs from conidia produced on cankers and other plant tissues carrying asymptomatic infections. This would be similar to many tree pathogens in the Botryosphaeriaceae, such as *Diplodia sapinea* (Bihon *et al*., 2011a, 2011b) and *Botryosphaeria dothidea* (Marsberg *et al*., 2017), which infect healthy plant tissues and remain dormant until environmental conditions or host susceptibility allow disease symptoms to develop. Studies showing that *T. gauchensis *and *T. zuluensis *can exist endophytically as latent pathogens in healthy plant tissues have yet to be conducted. However, the fact that *T. zuluensis *is found on seed capsules in the absence of symptoms (Jimu *et al*., 2016c), and that Kemler *et al*. (2013) found Teratosphaeriaceae to be one of the most common groups of fungi existing in healthy *Eucalyptus* tissues, provides strong evidence that these canker pathogens have a latent asymptomatic phase in their life cycle.

## Current Concerns

The spread of *T. gauchensis *and *T. zuluensis *to plantations across the world presents an immense risk to *Eucalyptus *forestry. Despite the known threats, pathogens continue to be introduced into new areas, illustrated by the recent emergence of *T. gauchensis *in Portugal and Zimbabwe (Jimu *et al*., 2015; Silva *et al*., 2015). The infection of *E. globulus *by *T. gauchensis* in Portugal is specifically alarming, as it represents a mild climate and a new host that usually only occurs in this mild climate. The expanded range of Teratosphaeria canker therefore increases the risk of adaptation to different environmental conditions.

The introduction of new genotypes to plantations already affected by the disease also remains concerning. The population size and genetic diversity of pathogens are regarded as important measures of their adaptability (McDonald and Linde, 2002; McDonald and McDermott, 1993); multiple introductions may increase this adaptability and, consequently, the ability to cause disease. The genetic diversity of *T. gauchensis *and *T. zuluensis *in certain parts of the world, especially China and Zimbabwe, implies that multiple genotypes have been introduced independently (Chen *et al*., 2011; Jimu *et al*., 2016b). In areas with a high genetic diversity, more than one genotype occurs on a single host (Chen *et al*., 2011; Jimu *et al*., 2016b). In addition, Asian and African populations of *T. zuluensis *and South American populations of *T. gauchensis *show evidence of recombination, despite the lack of a known sexual state in either stem canker pathogen (Cortinas *et al*., 2011; Jimu *et al*., 2016a).

For several years, *T. gauchensis *and *T. zuluensis *were known as species causing the same disease, but in distinct geographical locations. Both species, however, continue to spread and, in 2014, *T. gauchensis *and *T. zuluensis *were detected in the same *E. grandis *plantation in Uganda (Jimu *et al*., 2014). One year later, *T. gauchensis *was found to be widespread in the southern African country of Zimbabwe (Jimu *et al*., 2015), a region in which only *T. zuluensis *was previously known from South Africa, Mozambique and Malawi (Roux *et al*., 2005). Undoubtedly, anthropogenic movement of plant material to and amongst African countries has brought these two pathogens into close proximity (Fig. 1).

The consequences of virulent *T. gauchensis *and *T. zuluensis *strains infecting the same host simultaneously are unknown. One species may simply outcompete and replace the other, as has been observed between the Dutch elm disease pathogens *Ophiostoma ulmi *and *O. novo‐ulmi* (Brasier, 2001). Only the asexual forms of *T. gauchensis *and *T. zuluensis *are known and, in the absence of sexual reproduction, the opportunity for the acquisition of novel alleles is limited. History has shown that reproductive isolation in closely related allopatric fungi is often incomplete, and that contact between such species often leads to hybridization (Olson and Stenlid, 2002). This could be an ideal means for *T. zuluensis *and *T. gauchensis *to circumvent their apparent low rates of recombination.

The outcome of hybridization between two pathogens may be variable, ranging from unfit hybrids to a novel lineage that outcompetes its parents or establishes in a new niche (Olson and Stenlid, 2002). Examples of the latter situation include hybrids within the rust genus *Melampsora *(Newcombe *et al*., 2000; Spiers and Hopcroft, 1994) and the oomycete genus *Phytophthora* (Érsek and Nagy, 2008), which have led either to expanded host ranges or the collapse of host resistance (Olson and Stenlid, 2002). Between the extremes of unsuccessful to successful hybridization lies the possibility of introgression. Even short‐lived hybrids may backcross, acting as ‘genetic bridges’ that facilitate gene transfer between parent taxa (Brasier, 2001; Harrison and Larson, 2014). For example, despite the clear fitness advantage of *O. novo‐ulmi*, it has acquired vegetative compatibility genes from *O. ulmi *via introgression (Brasier, 2001). Advantageous characteristics are bound to introgress more easily as a result of favourable selection (Harrison and Larson, 2014) and, given ideal conditions, genes that influence important characteristics, such as pathogenicity, temperature sensitivity or host range, may be shared between *T. zuluensis *and *T. gauchensis*.

## Future Prospects

After the initial outbreak of Teratosphaeria canker in South Africa (Wingfield *et al*., 1996), the majority of susceptible *E. grandis *trees were replaced with resistant clones (Cortinas *et al*., 2010). *Teratosphaeria zuluensis *perpetuated in the susceptible clones that were not replaced (Cortinas *et al*., 2010), but Teratosphaeria canker became only a minor problem in the remainder of the plantation (Fig. 2e). Similarly, in Ethiopia, stands of healthy *E. camaldulensis *border infected *E. camaldulensis *stands, implying that they are resistant to *T. gauchensis *(Gezahgne *et al*., 2005). Both examples highlight the value of resistance breeding and selection in combating this disease.

Molecular genetic studies have played an important role in elucidating the taxonomy and geographical distribution of Teratosphaeria stem canker pathogens. Phylogenies based on DNA sequences have substantially improved our understanding of the taxonomy of these fungi (e.g. Cortinas *et al*., 2006c), and studies with microsatellite markers have unravelled their genetic diversity (or lack thereof) globally (e.g. Cortinas *et al*., 2010). However, little is known of their mode of infection, interaction with *Eucalyptus *hosts and the underlying basis of their specificity to stem rather than leaf tissues.

The genomics era has enabled many advances in the field of plant pathology (Aylward *et al*., 2017), as exemplified by the discovery of *T. zuluensis *as an endophyte and seed contaminant (Jimu *et al*., 2016c; Kemler *et al*., 2013). The application of ‘omics’ techniques to *T. zuluensis *and *T. gauchensis* will provide the next step towards addressing the current knowledge gaps, and will clearly accelerate the acquisition of new knowledge regarding these important tree pathogens. Both species are currently targeted for whole‐genome sequencing (GOLD projects Gp0311872 and Gp0311873; https://gold.jgi.doe.gov). These sequences will enable comparative genomics studies between the two stem pathogens, as well as their leaf‐associated relatives, for which several genomes are in the pipeline. The initial aims of these studies will be to determine the mating type systems of *T. zuluensis *and *T. gauchensis*, evaluate their risk of hybridization and discover which adaptations characterize their habitat switch from leaves to stems.

## Supporting information


**Fig. S1** ****Taxonomic history of *Teratosphaeria zuluensis* and *T. gauchensis*.

**Fig. S2** ****Culture morphology of *Teratosphaeria gauchensis* (row a, b) and *T. zuluensis* (row c, d) from above (row a, c) and below (row b, d). Cultures were grown on malt extract agar for 3 weeks at 26 °C, using an initial 5‐mm mycelial plug and 65‐mm Petri dishes.

**Text S1  **Phylogenetic methods and GenBank^®^ accession numbers used to construct Fig. 3.
