# Supplementary figures and images for: Teratosphaeria stem canker of Eucalyptus: two pathogens, one devastating disease
Source: Mol Plant Pathol. 2018 Nov 3;20(1):8–19. doi: 10.1111/mpp.12758 (PMC6430483; doi:10.1111/mpp.12758)

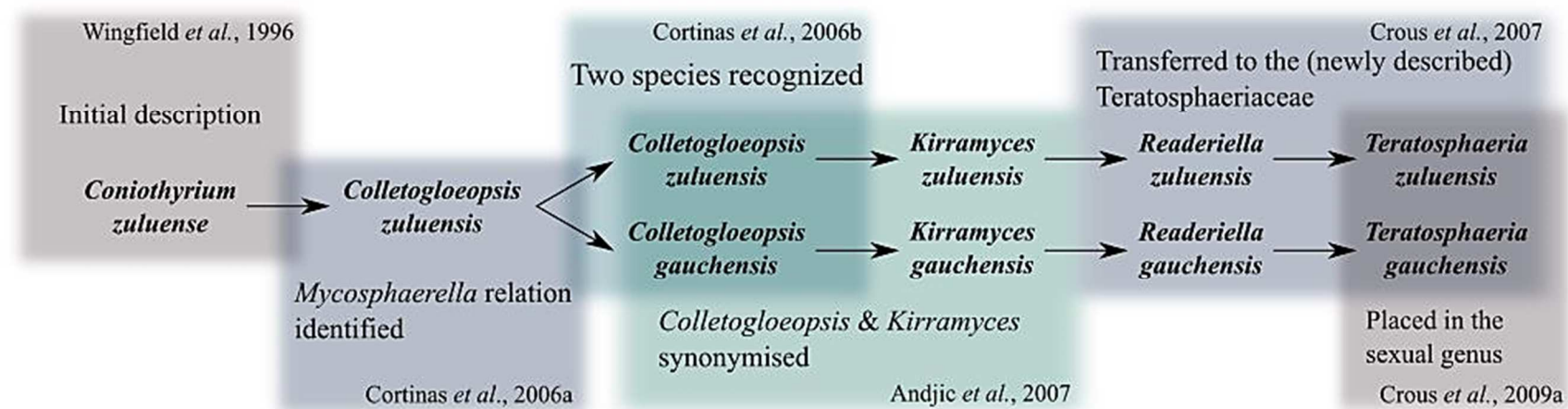

**Figure S1** Taxonomic history of *Teratosphaeria zuluensis* and *T. gauchensis*.

Supplement: Supplementary file 1 — Fig. S1 Taxonomic history of Teratosphaeria zuluensis and T. gauchensis. [file MPP-20-8-s001.pdf]
