# Supplementary material for: Teratosphaeria stem canker of Eucalyptus: two pathogens, one devastating disease
Source: Mol Plant Pathol. 2018 Nov 3;20(1):8–19. doi: 10.1111/mpp.12758 (PMC6430483; doi:10.1111/mpp.12758)
Supplement: Supplementary file 2 — Fig. S2 Culture morphology of Teratosphaeria gauchensis (row a, b) and T. zuluensis (row c, d) from above (row a, c) and below (row b, d). Cultures were grown on malt extract agar for 3 weeks at 26 °C, using an initial 5‐mm mycelial plug and 65‐mm Petri dishes. [file MPP-20-8-s002.pdf]

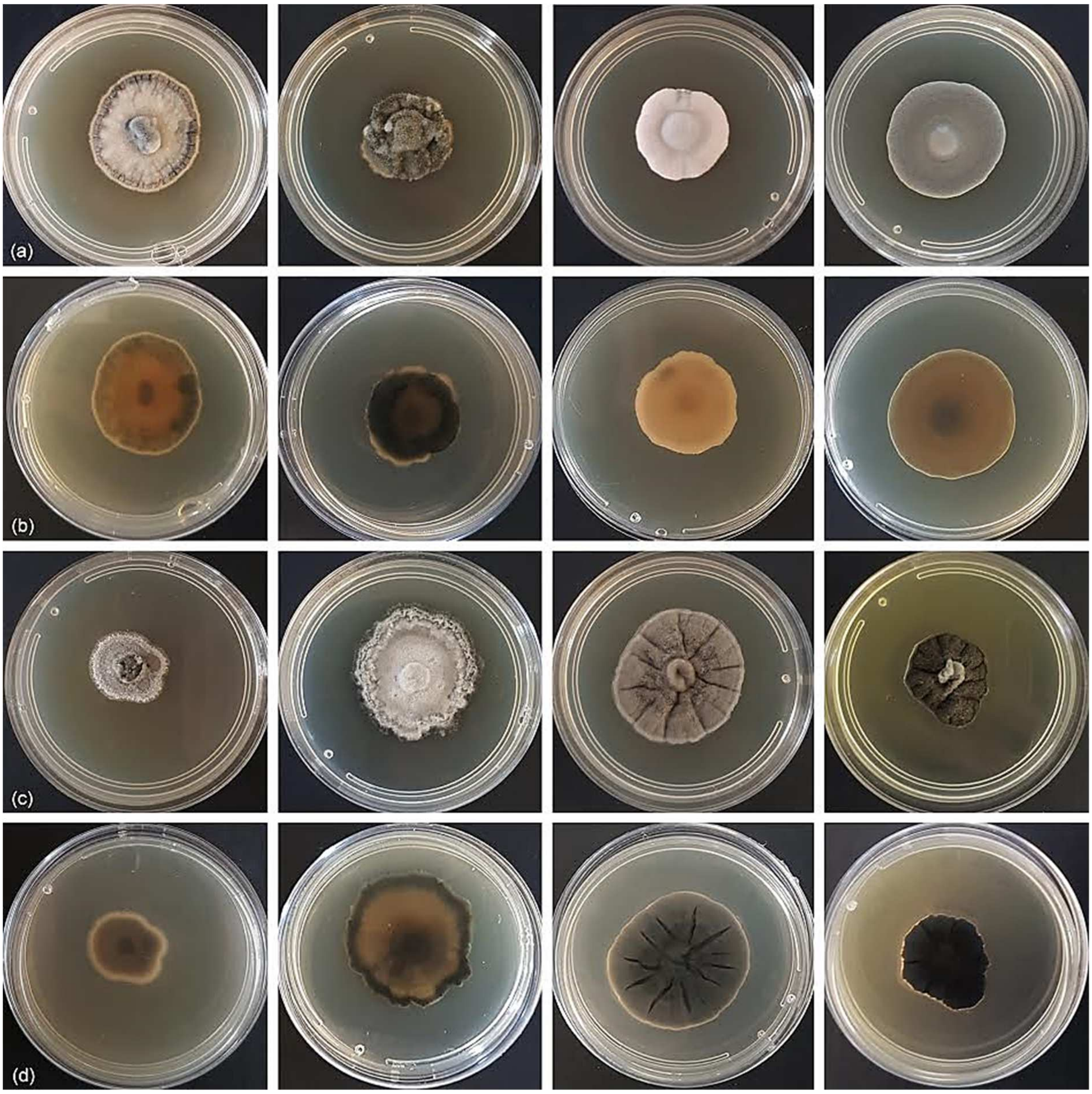

Figure S2 Culture morphology of *Teratosphaeria gauchensis* (row a-b) and *T. zuluensis* (row c-d) from above (row a, c) and below (row b, d). Cultures were grown on Malt Extract Agar for three weeks at 26°C, using an initial 5 mm mycelial plug and 65 mm petri dishes.
