# Supplementary material for: Teratosphaeria stem canker of Eucalyptus: two pathogens, one devastating disease
Source: Mol Plant Pathol. 2018 Nov 3;20(1):8–19. doi: 10.1111/mpp.12758 (PMC6430483; doi:10.1111/mpp.12758)
Supplement: Supplementary file 3 — Text S1 Phylogenetic methods and GenBank® accession numbers used to construct Fig. 3. [file MPP-20-8-s003.pdf]

Teratosphaeria phylogenetic analyses

Teratosphaeria isolates closely related to *T. zuluensis* and *T. gauchensis* were chosen based on the phylogeny of Quaedvlieg *et al.* (2014). The Beta-tubulin (Btub) and Elongation factor-1α (EF-1α) genes of 27 taxa (Table S1), including two *Readeriella* outgroup species, were aligned separately with MAFFT version 7 (Katoh and Standley, 2013) and the alignment cleaned with Gblocks 0.91b (Talavera and Castresana, 2007), using the default settings. For each gene region, maximum likelihood (ML) phylogenies were computed with PhyML 3.0 (Guindon *et al.*, 2010), enabling Smart Model Selection (Lefort *et al.*, 2017) by the Akaike Information Criterion (AIC) and performing 1000 bootstrap replicates to calculate branch support. The two ML gene trees were inspected visually for incongruence at nodes supported by at least 70 % bootstrap values in both trees. After confirming similar tree topologies for Btub and EF-1α, the ML analysis was repeated on the concatenated dataset (Fig. S3). Bayesian Inference on the concatenated dataset was performed with MrBayes v3.2.6 (Ronquist *et al.*, 2012), using the standard settings and five million generations of the Markov chains. To calculate the best model for each data partition, the nst option was set to "mixed" and a gamma distribution was included for both gene regions, as suggested by jModelTest 2.1.10 (Darriba *et al.*, 2012, Guindon and Gascuel, 2003). After a burn-in of 25% was applied, all ESS (estimated sample size) values, assessed in Tracer v1.6 (tree.bio.ed.ac.uk/software/tracer/), were greater than 660. Trees were viewed and exported with FigTree v1.4.3 (tree.bio.ed.ac.uk/software/figtree/). Fig. 3 (main text) reports the 50 % majority-rule Bayesian consensus tree (harmonic mean of posterior probabilities after burn-in = -4679.22) and includes the corresponding bootstrap values of the ML analysis.

Table S1 GenBank accession numbers of taxa used in the phylogenetic analysis.

**Species**	**Strain**[1]	**GenBank accession number**	
		**EF-1a**	**Btub**
Readeriella dimorphospora	CBS 120034 = CPC 12636; ET	KF903252	KF902956
R. dendritica	CBS 120032 = CPC 12709; ET	KF903251	KF902955
Teratosphaeria alboconidia	CBS 125004 = CPC 14598; ET	KF903283	KF902981
T. aurantia	CBS 125243 = MUCC 668; ET	KF903284	KF902984
T. blakelyi	CBS 120089 = CPC 12837; ET	KF903288	KF902988
T. callophylla	CBS 124584 = MUCC 700; ET	KF903289	FJ532003
T. considenianae	CPC 14057	KF903292	KF902991
T. corymbiae	CBS 124988 = CPC 13125	KF903293	KF902992
T. cryptica	CBS 110975 = CMW 3279 = CPC 936	KF903299	KF902998
T. destructans	CMW 44962 = SouthAfrica7	KT343575	KT343568
T. eucalypti	CPC 12552	KF903303	KF903002
T. foliensis	CBS 124581 = MUCC 670; ET	KF903311	KF903009
T. gauchensis	CBS 120303 = CMW 17331; ET	KF903315	KF903013
T. juvenalis	CBS 116427	KF903318	KF903016
T. majorizuluensis	CBS 120040 = CPC 12712; ET	KF903319	KF903017
T. molleriana	CBS 111164 = CMW 4940 = CPC 1214; ET of *Mycosphaerella molleriana*	KF903324	KF903021
T. nubilosa	CBS 116005 = CMW 3282 = CPC 937	KF903336	KF903033
T. ovata	CBS 124052 = CPC 14632	KF903345	KF903042
T. pseudoeucalypti	CBS 124577 = MUCC 607; ET	KF903349	KF903046
T. pseudonubilosa	CPC 13831	KF903350	KF903047
T. stellenboschiana	CBS 124989 = CPC 13767	KF903355	KF903052
T. epicoccoides (suttonii)	CBS 119973 = CMW 23439; ET of *Mycosphaerella obscuris*	KF903359	KF903055
T. toledana	CBS 113313 = CMW 14457; ET	KF903361	KF903058
T. veloci	CPC 14600	KF903363	KF903060

T. verrucosa	CBS 113621 = CPC 42; ET	KF903365	KF903062
T. viscidus	CBS 124992 = CPC 13306	KF903366	KF903063
T. zuluensis	CBS 120301 = CMW 17321; EET	KF903368	KF903064

[1] CBS = culture collection of the Westerdijk Fungal Biodiversity Institute, Utrecht, The Netherlands; CMW = culture collection of the Forestry and Agricultural Biotechnology Institute (FABI), University of Pretoria, South Africa; CPC: culture collection of Pedro Crous, housed at the Westerdijk Fungal Biodiversity Institute; ET = ex-type; EET = ex-epitype; MUCC = Murdoch University Culture Collection, Murdoch, Australia

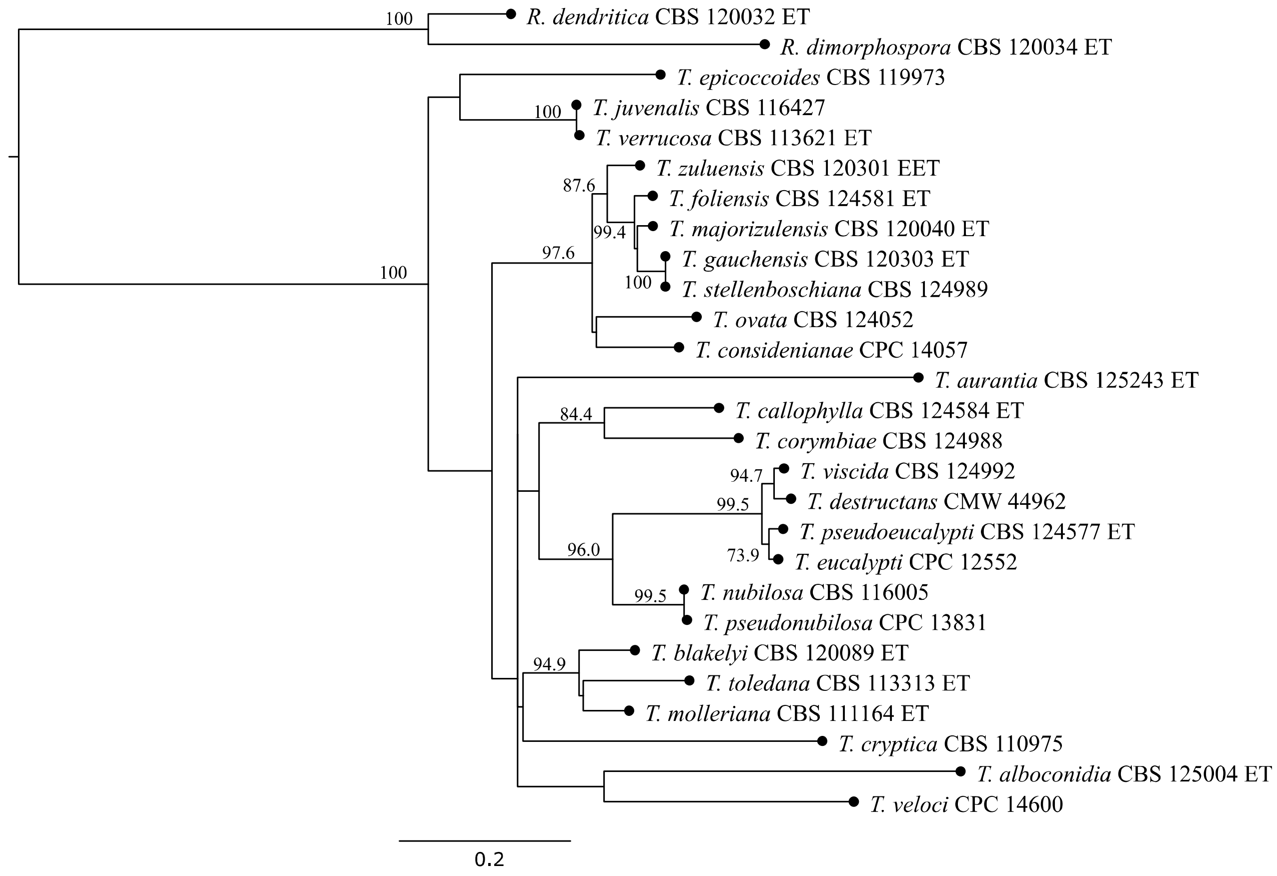

Figure S3 Maximum Likelihood phylogeny of the *Teratosphaeria* stem canker pathogens and related taxa. Values on branches represent bootstrap percentages. The log likelihood of the tree is -4619.70. ET = ex-type; EET = ex-epitype.

References

Darriba, D., Taboada, G. L., Doallo, R. and Posada, D. (2012) jModelTest 2: more models, new heuristics and parallel computing. *Nature Methods,* **9,** 772.

Guindon, S., Dufayard, J.-F., Lefort, V., Anisimova, M., Hordijk, W. and Gascuel, O. (2010) New algorithms and methods to estimate maximum-likelihood phylogenies: assessing the performance of PhyML 3.0. *Systematic Biology,* **59,** 307-321.

Guindon, S. and Gascuel, O. (2003) A simple, fast, and accurate algorithm to estimate large phylogenies by maximum likelihood. *Systematic Biology,* **52,** 696-704.

Katoh, K. and Standley, D. M. (2013) MAFFT multiple sequence alignment software version 7: improvements in performance and usability. *Molecular Biology and Evolution,* **30,** 772-780.

Lefort, V., Longueville, J.-E. and Gascuel, O. (2017) SMS: Smart model selection in PhyML. *Molecular Biology and Evolution,* **34,** 2422-2424.

Quaedvlieg, W., Binder, M., Groenewald, J. Z., Summerell, B. A., Carnegie, A. J., Burgess, T. I., ***et al.*** (2014) Introducing the Consolidated Species Concept to resolve species in the *Teratosphaeriaceae*. *Persoonia,* **33,** 1-40.

Ronquist, F., Teslenko, M., Van Der Mark, P., Ayres, D. L., Darling, A., Höhna, S., ***et al.*** (2012) MrBayes 3.2: efficient Bayesian phylogenetic inference and model choice across a large model space. *Systematic Biology,* **61,** 539-542.

Talavera, G. and Castresana, J. (2007) Improvement of phylogenies after removing divergent and ambiguously aligned blocks from protein sequence alignments. *Systematic Biology,* **56,** 564-577.
